# Determination of the Toxic Effects of Heavy Metals on the Morpho-Anatomical Responses of the Leaf of *Typha latifolia* as a Biomonitoring Tool

**DOI:** 10.3390/plants13020176

**Published:** 2024-01-09

**Authors:** Nedjma Mamine, Nedjoud Grara, Fadila Khaldi, Viviana Maresca, Khaoula Aouaichia, Adriana Basile

**Affiliations:** 1 Department of Biology, Faculty of Life and Natural Science, University of Mohamed Cherif Messaadia, Souk Ahras 41000, Algeria; nedjmamine00@gmail.com; 2 Department of Biology, Faculty of Nature, Life Sciences, Earth and Universe Sciences, University 8 May 1945, P.O. Box 401, Guelma 24000, Algeria; 3 Laboratory of Science and Technology of Water and Environment, University of Mohamed Cherif Messaadia, Souk Ahras 41000, Algeria; khaldifad@yahoo.fr (F.K.); aouaichia.khaoula@gmail.com (K.A.); 4 Department of Biology, University of Naples Federico II, 80126 Naples, Italy; viviana.maresca@unina.it

**Keywords:** Lake Burgas, constructed wetland, trace metals, phytoremediation, microscopic analysis

## Abstract

*Typha latifolia* leaves act as sensitive barometers for trace heavy metal pollution, as revealed by their pronounced anatomical responses in a constructed wetland. Monthly water samples and *Typha latifolia* leaf tissue were collected over three consecutive months in 2018 from the Burgas Lake wetlands (Taoura), northeast Algeria. While physical and chemical parameters improved after treatment, atomic absorption spectrometry (Perkin Elmer A Analyst 800 AAS) detected persistent trace levels of cadmium, chromium, and lead in both the treated water and leaf tissue, highlighting the need for continued phytoremediation efforts. Microscopic examination of leaf tissue exposed to these metals revealed distinct anatomical adaptations, including shrunken vascular bundles, altered cell shapes, and stomatal closure. These findings underscore *Typha latifolia*’s effectiveness in accumulating heavy metals and its potential as a highly sensitive biomonitor for persistent pollution in lake ecosystems.

## 1. Introduction

The escalation of demographic growth alongside rapid industrial development has led to severe environmental hazards. Human activities, such as industrial processes, mining, gas emissions, pesticide application, and waste production, have resulted in substantial pollution of soil, water, and atmospheric ecosystems [1,2]. Currently, aquatic environments harbor a wide array of xenobiotic and anthropogenic chemicals [3,4], which, either singly or in mixtures, pose threats to aquatic organisms due to their detrimental effects [5,6].

Heavy metals pollution presents a critical environmental challenge due to its non-biodegradability and hazardous nature [7]. Heavy metals have a tendency to accumulate and migrate within soil environments, potentially being absorbed by plants through their root systems. This accumulation can compromise ecosystem safety, posing threats to animals, plants, and human health. Elevated metal concentrations in plants can hinder chlorophyll production, heighten oxidative stress, and weaken stomatal resistance [8].

Noteworthy heavy metals such as cadmium, copper, lead, chromium, and mercury are significant environmental pollutants, especially in areas characterized by a strong anthropogenic impact. Trace amounts of these metals in the atmosphere, soil, or water can pose serious threats to all organisms. Bioaccumulation of heavy metals, particularly in the food chain, can be highly detrimental to human health. Human exposure to heavy metals occurs primarily through inhalation and ingestion, with ingestion being the predominant route (leading to endocrine system damage, immune impact, neurological disorders, and cancer) [9].

At the cellular level, toxic metal ions induce oxidative stress by generating reactive oxygen species (ROS), thereby causing DNA alterations, perturbing protein functionality, disrupting nutrient homeostasis, and impacting membrane and protein functions [10,11].

In Algeria, as in numerous African and global regions, various studies have evaluated the efficacy of constructed wetlands in wastewater treatment [12,13]. Monitoring and safeguarding the physicochemical and biological quality of water in aquatic ecosystems necessitate efforts to prevent and control metal pollution to mitigate its impact on water resources, biodiversity, food webs, and human health [14,15]. The integration of artificial wetlands (CW) within natural aquatic ecosystems presents a promising strategy for eco-remediation.

Recent advances have explored phytoremediation in artificial filter marshes as a technology to remove metallic pollutants, employing a combination of physical, chemical, and biological processes like sedimentation, precipitation, adsorption to soil particles, uptake by plant tissues, and transformation by microorganisms [16].

While several studies have noted the tolerance of naturally growing cattails to varying concentrations of heavy metals, such as *Typha latifolia* [13,14,15,16,17], *Typha domingensis*, and *Typha angustifolia* [18,19], the focus on examining treatment efficiency often revolves around physicochemical parameters [13,14,15,16,17,18,19,20]. Few studies have delved into assessing environmental stressors’ impact at the morphological and anatomical levels.

Helophytes, due to their ability to accumulate pollutants, serve as valuable ecological indicators of metal contamination in aquatic ecosystems. These biological tools naturally signal environmental pollution and the bioavailability of toxic substances [21].

The macrophyte *Typha latifolia* stands out as a prime model organism for studying pollutant toxicity in raw wastewater. Its well-documented capacity to accumulate significant concentrations of pollutants like cadmium, chromium, and lead in both subterranean and aerial tissues makes it a highly relevant choice [14].

This work aims to evaluate the effects, induced by pollutants present in raw wastewater, on the anatomical markers of the leaves of *T. latifolia*, a purifying macrophyte species in Lake Burgas (Taoura), northeastern Algeria. These macrophytes, serving as ecotoxicology models and biosurveillance tools in freshwater resources, can effectively function as biological indicators amidst environmental pressures. Additionally, this study determines the physicochemical parameters of this artificial wetland to evaluate wastewater treatment efficacy.

## 2. Results and Discussion

### 2.1. Physico-Chemical Characterization of Burgas Lake Waters

Table 1 shows the average values of measured parameters of Burgas Lake waters during the study period.

#### 2.1.1. Evolution of Temperature: T °C

Wetland water temperature ranged from 15.3 °C to 22.6 °C (Table 1), falling below the 30 °C limit for direct environmental discharge and wastewater irrigation [22,23]. Though technically acceptable for discharge, these fluctuations may nonetheless influence pollutant uptake by *T. latifolia* due to altered microbial activity and nutrient dynamics. Higher temperatures can favor organic pollutant degradation while also stressing the plants. pH within the recorded range (7.33–7.97) may further impact the bioavailability of metals and inorganic compounds for plant uptake.

#### 2.1.2. Evolution of Hydrogen Potential: pH

The hydrogen potential, an indicator of pollution, fluctuates due to the nature of effluents: basic (cooking, washing) or acidic (acetic acids, chlorine derivatives) [15].

The recorded pH range in Lake Burgas (7.33–7.97) falls within acceptable environmental limits; even slight fluctuations within this range can influence the ability of *T. latifolia* to assimilate pollutants. Studies have shown that pH variations can alter the speciation and bioavailability of metals and inorganic compounds, affecting their uptake by plants [24,25]. For instance, a shift towards a more acidic pH can increase the solubility and mobility of certain heavy metals, potentially making them more accessible to *T. latifolia* for phytoremediation [26,27]. Conversely, a higher pH might favor the formation of insoluble metal complexes, limiting their plant uptake [28]. Therefore, understanding the complex interplay between pH fluctuations and metal speciation is crucial for optimizing the efficiency *of T. latifolia* in pollutant removal within constructed wetlands.

#### 2.1.3. Evolution of Electrical Conductivity (EC)

Despite falling within the FAO’s permissible range (0–3000 µs/cm) [29], the measured electrical conductivity (EC) in Lake Burgas (1236.56–1563.23 µs/cm) exceeds the Algerian standard for irrigation water (<2000 µs/cm) [22]. This elevated EC, likely due to the organic load [30], could pose challenges for conventional irrigation. However, *T. latifolia* is a robust aquatic plant that thrives in such environments, showcasing remarkable capabilities in both pollutant removal and salinity tolerance [31].

#### 2.1.4. Evolution of Turbidity

Water clarity, measured as turbidity, plays a crucial role in the health and functionality of aquatic ecosystems. In Lake Burgas, it fluctuated considerably throughout the study period, ranging from 1.19 to 8.5 NTU (Table 1). This variability likely stems from the influx of finely divided suspended matter, including clay, silt, organic matter, and nitrogen compounds, through discharged effluents [15,16,17,18,19,20,21,22,23,24,25,26,27,28,29,30,31,32]. Fortunately, aquatic plants like *T. latifolia* can act as natural filters, effectively reducing turbidity. Their dense root systems and fibrous tissues capture suspended particles, allowing for gradual sedimentation and improved water clarity. This filtration capacity plays a vital role in maintaining a healthy ecosystem by promoting light penetration, oxygen availability, and overall water quality, highlighting the significant contribution of aquatic plants in managing turbidity fluctuations.

#### 2.1.5. Evolution of the Inorganic Load

Analysis of heavy metals in the wetland waters of Lake Burgas revealed the presence of Cr, Co, and Cd in low concentrations at the outlet [33] (Table 1). However, due to the short residence time, which limits the ability of phytoextraction to effectively remove and concentrate these pollutants in the root and aerial parts, phytoextraction is considered a minor factor in inorganic pollutant reduction [14,15,16,17,18,19,20,21,22,23,24,25,26,27,28,29,30,31,32,33,34]. The contamination of aquatic ecosystems with heavy metals from urban and industrial effluents poses a serious threat to human health, as these metals can be absorbed by vegetables and enter the food chain, causing bio-concentration at each higher trophic level [35,36].

### 2.2. The Effect of Environmental Pollution on the Anatomical Responses of T. latifolia

**Figure 1:**
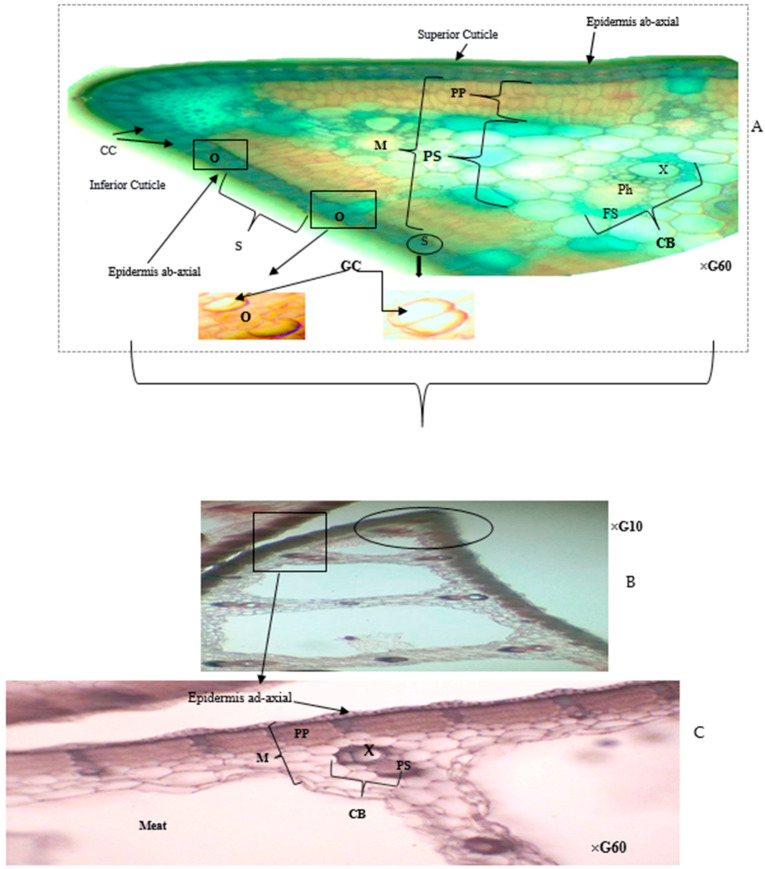
Paradermal sections of *T. latifolia* leaves (Witness) (**A**–**C**). CB: Conducting Bundles; CC: Companion Cells; FS: Fascicular Sheath; GC: Guard Cell; M: Mesophyll; S: Stomata; PS: Parenchyma of Spongy; PP: Palisade Parenchyma; O: Ostiole; Ph: Phloem; X: Xylem.

Examination of cross-sections from control and *T. latifolia* leaves exposed to raw sewage initially focused on identifying the control histological organization (Figure 1). This revealed that the leaf tissue of *T. latifolia* is composed of two cuticles: one on the upper surface and another on the lower surface. Each cuticle acts as a thin protective film covering the entire leaf epidermis. Subsequent microscopic observation of the epidermis, a single layer of generally elongated cells covering the leaf, further confirmed its dorsoventral anatomy: the upper (adaxial) and lower (abaxial) surfaces. The closely knit epidermal cells form a boundary between the plant and the external environment (Figure 1A,B).

**Figure 2:**
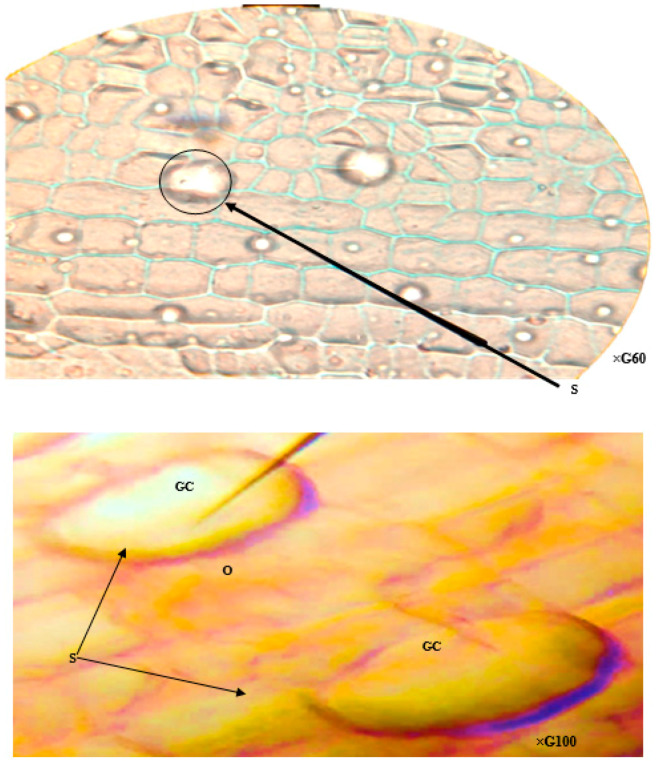
Stomatal characteristics of *T. latifolia* leavs (Witness). GC: Guard Cell; S: Stomata; O: Ostiole.

The abaxial face specifically exhibits unique epidermal structures: the epistomatic stomata. These structures comprise a single stomatal type, the anomocytic variety, which features two bean-shaped guard cells that are thicker on the internal side and frame an opening called the ostiole. These guard cells are often accompanied by companion cells, lacking chloroplasts, with which they share intimate contact via their external faces (Figure 1A and Figure 2).

Moving inward from the epidermal layers, the mesophyll, constituting the bulk of the leaf interior, comes into view (Figure 1A). This non-homogeneous tissue is further divided into two parts:

The chlorophyllous palisade parenchyma, located directly beneath the upper epidermis, consists of a single layer of elongated and tightly packed cells (Figure 1C).

In contrast, the lacunar (spongy) chlorophyll parenchyma toward the lower epidermis features relatively rounded cells with fewer chloroplasts and is interspersed with large air spaces.

Monocot leaf inner tissues are characterized by parallel veins reinforced with bundles of elongated sclerenchyma fibers, providing high tensile strength. These veins comprise conducting bundles of xylem and phloem, with the xylem typically positioned on the upper side of the veins and the phloem on the lower side. A surrounding set of cells called the fascicular sheath encloses the veins, which are then further surrounded by mesophyll cells.

**Figure 3:**
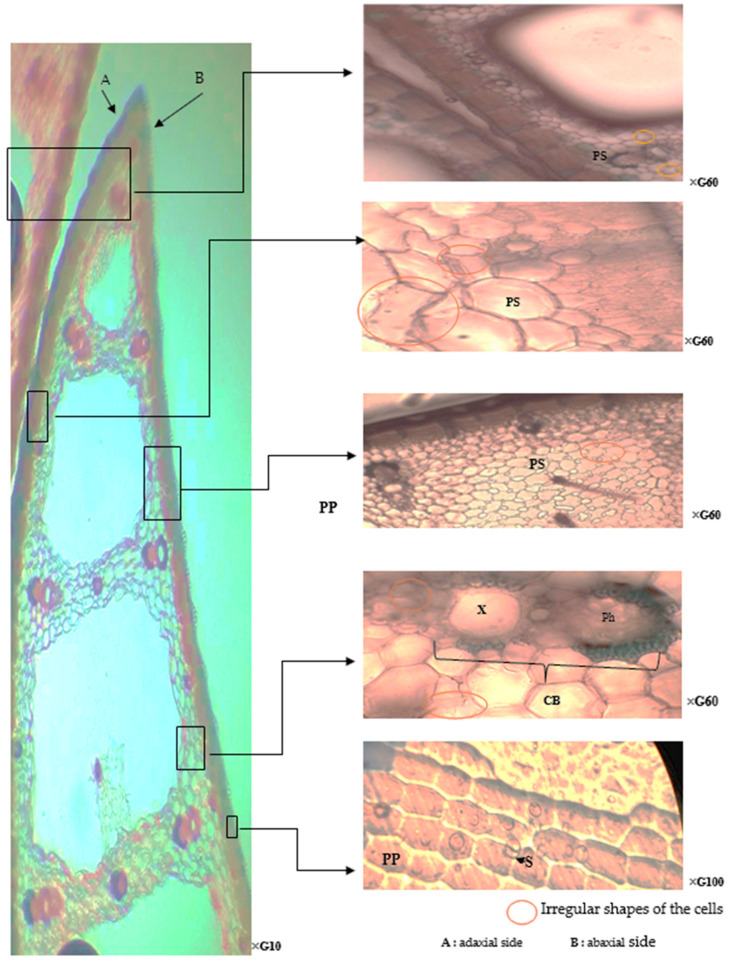
Paradermal sections of *T. latifolia* leaves (under wastewater stress). S: Stomata; PS: Parenchyma of Spongy; PP: Palisade Parenchyma; CB: Conducting Bundles; Ph: Phloem; X: Xylem.

Microscopic observation of *T. latifolia* leaves exposed to raw sewage toxicity revealed pronounced anatomical alterations compared to the control leaves. Notably, the spongy parenchyma tissue exhibited irregular cell shapes and structural modifications (Figure 3).

### 2.3. Cytotoxic Effects of Heavy Metals and Bioremediation Potential

The presence of heavy metals (Cr, Cd, and Pb) in raw wastewater is evidenced by their cytotoxic effects. These compounds induce oxidative damage through membrane lipid destruction. Studies by Amir et al. [34] and Mamine et al. [14] have demonstrated that hyperaccumulating and heavy metal-tolerant plants like *T. latifolia* hold promise for bioremediation applications.

Histological analysis revealed alterations in the conductive vessels of leaves exposed to raw wastewater compared to control leaves. These changes included shrinkage of vessel cell size and slight deformations in the xylem. This phenomenon is likely associated with an initial biological response to the stress induced by heavy metals encountered during the hydraulic operation of planted systems. Iqbal et al. [36] proposed that anatomical changes in *T. latifolia* xylem vessel elements might be triggered by wall material deposition. This could represent a potential strategy to restrict water flow, thereby limiting the mobility and transport of absorbed metals and protecting cellular components from their toxic effects. Furthermore, Kong et al. [37] observed increased Cd concentration at the xylem level of hydroponically grown Salix matsudana Koidz in response to elevated production of phytochelatins (e.g., proline). These chelating agents bind Cd, preventing its deleterious morphological impacts caused by Cd-induced reactive oxygen species (ROS).

Heavy metals pose a significant threat to the health of aquatic ecosystems. Unlike organic chemicals, they cannot be readily removed through natural processes of degradation into less toxic compounds [38]. This characteristic presents unique challenges for their removal from contaminated water.

The utilization of cattail macrophytes (*T. latifolia*) in phytoremediation strategies for aquatic ecosystems offers a unique tool for the remediation of Burgas Lake. These plants can accumulate heavy metals from contaminated water via their roots [14], with subsequent transfer to rhizomes and aerial parts (stems, leaves) [39,40]. Therefore, *T. latifolia* may play a crucial role in mitigating metal toxicity within the aquatic environment.

### 2.4. The Paradermal Sections of the Leaves

Examination of peridermic sections from polluted site leaves revealed the presence of epistomatic stomata solely on the abaxial surface. These stomata exhibited a single type, the anomocytic variety, as visualized in Figure 1A and Figure 2.

Notably, the observed homogeneity in stomatal features contrasted with the expected variety. This finding can be attributed to the inherent characteristics of cattail aquatic macrophyte stomata, reflecting adaptations for efficient carbon acquisition from the surrounding environment.

Despite the anatomical uniformity, a notable alteration in stomatal behavior was observed compared to control samples. Figure 3 demonstrates the closure of most stomata in polluted site leaves, suggesting that *T. latifolia* tolerates heavy metal exposure by regulating water loss, potentially through stomatal control mechanisms [41].

It is important to remember that stomata are specialized epidermal cells crucial for CO_2_ absorption, while simultaneously releasing oxygen and water vapor. These vital structures play a fundamental role in the process of photosynthesis [42].

Doheny-Adams et al. [43] established a link between structural changes in stomata and both genetic and environmental factors. Additionally, Brodribb et al. [44] demonstrated that plants exhibit a degree of homeostasis in leaf water content. This mechanism protects photosynthetic and xylem tissues from damage while maintaining efficient resource allocation. The tight coordination between xylem-mediated water delivery and stomatal-regulated water loss plays a key role in achieving this homeostasis.

## 3. Materials and Methods

### 3.1. Presentation and Description of the Study Area

The present study was conducted at Burgas Lake, a fragile wetland situated near Taoura (ancient Thagora) in the city of Souk Ahras, northeastern Algeria. Threatened by urban expansion, this lake constitutes a vital open water surface (FW-CW) featuring submerged vegetation and stands of *T. latifolia*. Notably, *T. latifolia* exhibits roots thriving in the waterlogged substrate while its reproductive and vegetative organs remain above the water.

**Figure 4:**
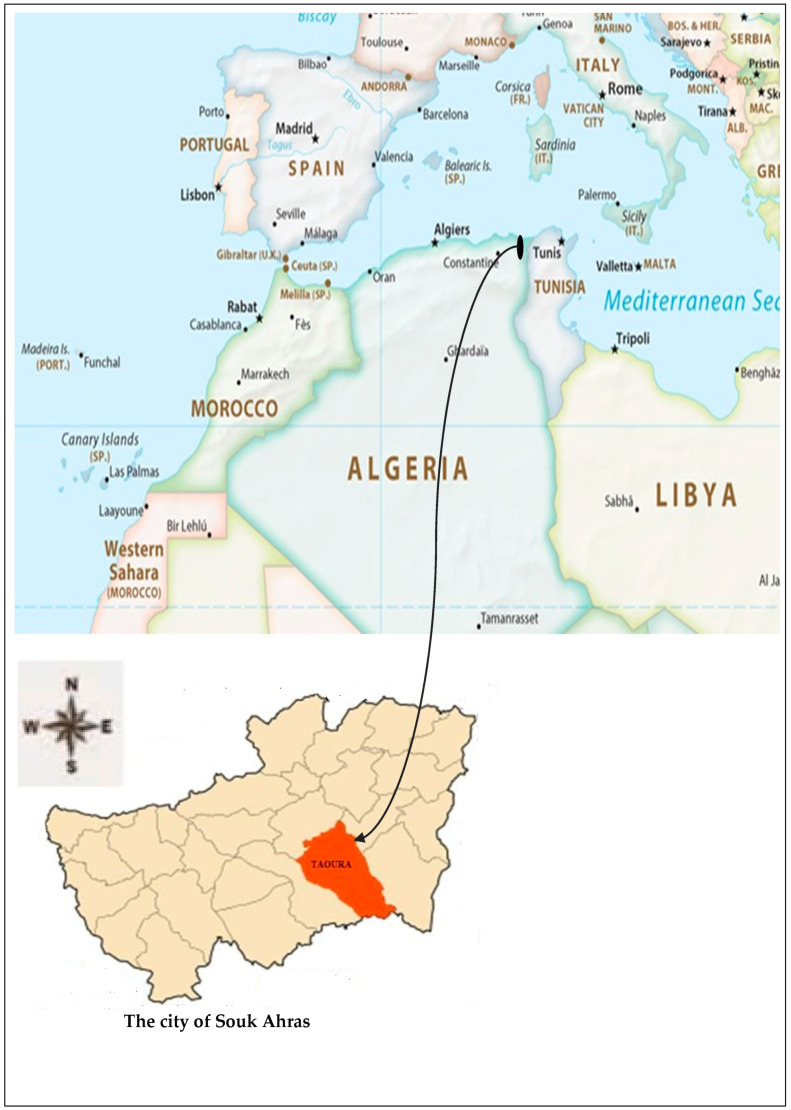
The geographical location of the study area: Burgas lake (Taoura city of Souk Ahras), northeastern Algeria [45].

Burgas Lake, boasting the title of Souk Ahras’ largest natural lake, lies approximately 20 km southeast of the city. Its location near Taoura, revered as Thagora in ancient times, further enriches its cultural significance. This lake serves as a cherished stopover for diverse bird species during their migratory journeys. The prevailing climate in the region can be categorized as arid to semi-arid, with average temperatures fluctuating between a 2 °C minimum and a 37 °C maximum (Figure 4).

**Figure 5:**
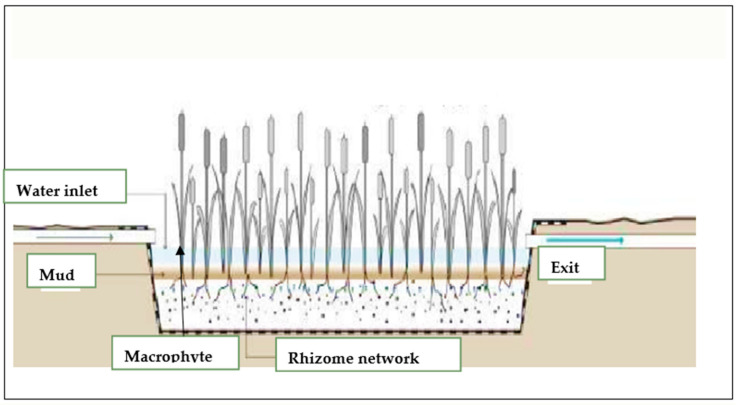
Diagram of operation of constructed wetlands with open water surface (FW-CW).

Figure 5 presents a schematic illustration of the treatment processes within a constructed wetland. As wastewater traverses the wetland, it undergoes a series of physical, chemical, and biological transformations that effectively remove pollutants. Notably, sedimentation, filtration, and microbiological degradation serve as the primary mechanisms involved. These processes efficiently eliminate ammonia, suspended solids, and various organisms. Additionally, nitrogen removal demonstrates high efficiency, while phosphorus removal exhibits less efficacy. Constructed wetlands are extensively employed in the tertiary treatment of both municipal wastewater and stormwater runoff.

### 3.2. Water Sampling and Analysis Methods 

This work is based first on a survey of the functioning of the Burgas Lake wetlands that was conducted in the months of April to June during the year 2018. During this period, we performed weekly water sampling at the outlet of the Burgas Lake wetland. The operation was performed manually using a small container that was then transferred into bottles before taking the sample to perform the appropriate analyses. The samples were filled into clean polyethylene bottles with Teflon caps, and rinsed at the time of use with the water to be examined [46]. These samples were subject to measurements of the parameters in situ, which included the hydrogen potential (pH), temperature (T °C), electrical conductivity (EC), turbidity (Turb), and dissolved oxygen (O_2_) of the water. These were determined using a multiparameter field brand Consort C 562; laboratory analysis focused on metal composition via atomic absorption spectrometry (Perkin Elmer A Analyst 800 AAS, American Laboratory Trading, East Lyme, CT, USA) for Hg, Cd, Pb, and Cr.

### 3.3. Collection and Sampling of Plants

**Figure 6:**
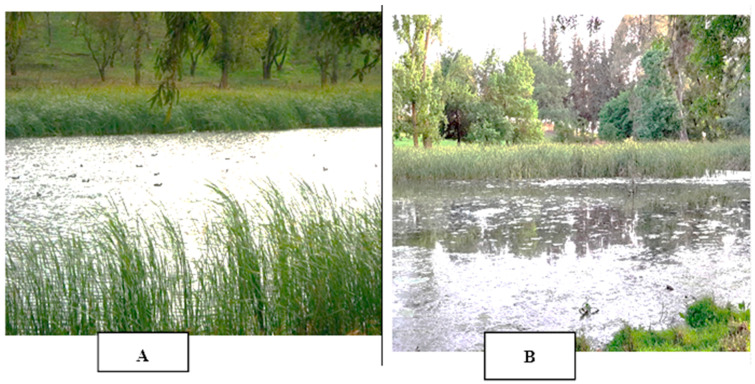
Location of the collection site the cattails *T. latifolia* collected from Burgas lake: (**A**) unpolluted area and (**B**) polluted area.

The investigation of plant communities within Burgas Lake necessitated the selection of two distinct sampling locations:

Polluted Area: This site, readily identifiable in Figure 6B, exhibited a high abundance of aquatic *T. latifolia* plants. This is indicative of the area’s polluted status.

Unpolluted Area (Control): This reference site, showcased in Figure 6A, represented an unpolluted region of the lake and served as a crucial control for comparison. Monthly sampling was conducted throughout April, May, and June at each location. During each sampling event, eight young plants were carefully collected for further analysis.

### 3.4. Study Species Description

The species *T. latifolia* is a perennial emergent macrophyte that often dominates the upper littoral zone of eutrophic lakes and the edges of rivers in temperate and subtropical areas [47]. This species produces up to 3 m high linear leaves and an extensive system of horizontal rhizomes. Cattails can grow on organic, highly reduced sediments, as well as on acidic sites with high concentrations of reduced metal ions in the interstitial water [48]. The basis for treatment with these rooted helophytes is particularly based on the following facts:

- They are plants with horizontal and vertical rhizomes that provide support for the growth of bacteria and the filtration of particulate substances [49].
- The rhizome also provides, together with the roots, high soil permeability and a large soil–wastewater contact area [50].
- They can concentrate heavy metals, absorb more nutrients than they need, and neutralize extreme pH [51,52].
- They can transmit oxygen from the leaves through the stems into the rhizosphere [16,17,18,19,20,21,22,23,24,25,26,27,28,29,30,31,32,33,34,35,36,37,38,39,40,41,42,43,44,45,46,47,48,49,50,51,52,53].

#### 3.4.1. Selection and Preparation of Biological Material

This study focused on young leaves of the rooted helophyte *T. latifolia*. These samples were carefully chosen to ensure consistent representation of the plant community. To prevent desiccation during transport and storage, the collected leaves were promptly immersed in distilled water.

#### 3.4.2. Realization of Anatomical Sections and Staining

At the level of the leaf structure, sections were prepared using the freehand manual technique. Subsequently, the subjects were stained according to the double staining technique (carmine-green) as described in the following steps [54]:

- Sectioning: Using a razor blade, thin sections of the organs under study were carefully cut. The thinnest and most suitable slices were then selected for further processing.
- Bleaching: The selected sections were placed in a watch glass containing bleach. This step aimed to dissolve the cellular contents while preserving the cell walls made of pectin and cellulose.
- Rinsing: To remove excess bleach, the sections were transferred to a second watch glass filled with distilled water.
- Mordanting: To eliminate any remaining bleach and prepare the tissues for staining, the sections were immersed in a 1% acetic acid solution for two minutes. This step acted as a cell mordant, enhancing the staining process.
- Staining: The sections were then stained in a fourth watch glass containing a mixture of equal parts Congo red and carmine alum. Congo red stains dead, lignified tissues red, while carmine alum stains living tissues and intensifies the red color with cellulose.
- Dehydration: After staining, the sections were placed in 70% alcohol. This step replaced the water within the cells, helping to preserve the obtained sections.

#### 3.4.3. Visualization and Photography

- Mounting: The prepared sections were carefully placed between a slide and a coverslip, with a drop of glycerin added to enhance clarity.
- Microscopic Examination: The mounted sections were then positioned under the optical microscope for visualization.
- Documentation: Simultaneously with the microscopic examination, photographs of the histological sections were captured using an OPPO A53 camera (OPPO Electronics Corp., Ltd., Dongguan, China). These images allow for further analysis and record-keeping of the observed features.

### 3.5. Statistical Analysis

Data processing and analysis were conducted using the statistical software package Statistica^®^ 8.0 [55]. Results were expressed as the mean ± standard deviation for each studied physico-chemical variable. Statistical procedures adhered to the recommendations outlined by Dagnelie [55].

## 4. Conclusions

In conclusion, *T. latifolia* in the Burgas Lake wetlands demonstrated noticeable morphological and anatomical changes in response to heavy metal stress from municipal wastewater. These changes, including shrunken vascular bundles, irregular spongy parenchyma cell shapes, and slight xylem deformations, offer valuable insights into the plant’s physiological response to metal contamination. By quantifying and correlating these alterations with known metal concentrations, *T. latifolia* can be utilized as a sensitive bioindicator for monitoring and assessing heavy metal pollution in aquatic environments. This approach offers several advantages: it is non-destructive, cost-effective, and potentially provides real-time information on metal pollution levels. Future research should focus on establishing robust correlations between specific anatomical changes and metal concentrations, enabling the development of standardized bioindicator indices for *T. latifolia*. Additionally, research on metal bioavailability in other aquatic macrophytes could broaden the range of available bioindicators for comprehensive environmental monitoring.

## Figures and Tables

**Table 1:** General statistics of the measured physico-chemical parameters.

Variable Physico-Chemical		Number of Samples	Minimum Values	Maximum Values	Mean Values ± Standard Deviation
Parameters analyzed in the laboratory	Hg (ppm)	12	0.001	0.060	0.015 ± 0.01
	Cd (ppm)	12	0.004	0.07	0.02 ± 0.02
	Cr (ppm)	12	0.02	0.06	0.04 ± 0.01
	Pb (ppm)	12	0.001	0.005	0.003 ± 0.056
Parameters measured in situ	Turbidity (NTU)	12	90.2	188	73.81 ± 12.2
	Oxygen dissolved (O_2_) (mg/L)	12	1.67	3.37	2.45 ± 0.87
	pH	12	7.33	7.97	7.69 ± 1.18
	T °C	12	15.3	22.56	13.43 ± 1.54
	EC (μs/cm)	12	1236.56	1563.23	1385.11 ± 152.87

Cd: Cadmium; Cr: Chromium; Pb: Lead; pH: hydrogen potential; T: Temperature; EC: Electrical conductivity.

## Data Availability

All data are included in this manuscript.
